# Influence of Matrices on 3D-Cultured Prostate Cancer Cells' Drug Response and Expression of Drug-Action Associated Proteins

**DOI:** 10.1371/journal.pone.0158116

**Published:** 2016-06-28

**Authors:** Rasheena Edmondson, Audrey F. Adcock, Liju Yang

**Affiliations:** Department of Pharmaceutical Sciences, Biomanufacturing Research Institute and Technology Enterprise (BRITE), North Carolina Central University, Durham, NC 27707, United States of America; Thomas Jefferson University, UNITED STATES

## Abstract

This study investigated the effects of matrix on the behaviors of 3D-cultured cells of two prostate cancer cell lines, LNCaP and DU145. Two biologically-derived matrices, Matrigel and Cultrex BME, and one synthetic matrix, the Alvetex scaffold, were used to culture the cells. The cell proliferation rate, cellular response to anti-cancer drugs, and expression levels of proteins associated with drug sensitivity/resistance were examined and compared amongst the 3D-cultured cells on the three matrices and 2D-cultured cells. The cellular responses upon treatment with two common anti-cancer drugs, Docetaxel and Rapamycin, were examined. The expressions of epidermal growth factor receptor (EGFR) and β-III tubulin in DU145 cells and p53 in LNCaP cells were examined. The results showed that the proliferation rates of cells cultured on the three matrices varied, especially between the synthetic matrix and the biologically-derived matrices. The drug responses and the expressions of drug sensitivity-associated proteins differed between cells on various matrices as well. Among the 3D cultures on the three matrices, increased expression of β-III tubulin in DU145 cells was correlated with increased resistance to Docetaxel, and decreased expression of EGFR in DU145 cells was correlated with increased sensitivity to Rapamycin. Increased expression of a p53 dimer in 3D-cultured LNCaP cells was correlated with increased resistance to Docetaxel. Collectively, the results showed that the matrix of 3D cell culture models strongly influences cellular behaviors, which highlights the imperative need to achieve standardization of 3D cell culture technology in order to be used in drug screening and cell biology studies.

## Introduction

Cell-based assays have been important tools in the drug discovery process to provide the first choice for drug compound screening. The global market for cell-based assays is continuously increasing. Many pharmaceutical and biotech companies are using cell-based assays as alternatives to biochemical assays, and *in vitro* testing for identification and validation of targets, screening for efficacy and safety, and monitoring cell-based activities. The cultured cells are the key element of such techniques. Undoubtedly, traditional two-dimensional (2D) cell culture, in which cells are grown on a flat rigid substrate to form a monolayer, has proven to be a valuable tool for cell-based assays. For years, 2D cell culture has been used for compound screening, toxicity studies, as well as for studying many areas of cancer cell biology such as the transition of normal cells to cancer cells, the differential expression of genes and proteins associated with tumor development and prognosis, and anti-cancer treatments [1, 2]. Although the time-honored 2D cell culture has made invaluable contributions to drug discovery and the understanding of cancer cell biology, its limitations have been increasingly recognized in recent years. Considering the *in vivo* environment, almost all cells are surrounded by neighboring cells and/or the extracellular matrix (ECM), and their interactions are all in a three-dimensional (3D) fashion. Obviously, not being able to mimic the natural 3D environment of cells is a limitation of traditional 2D culture technique, and as a result, 2D cell culture tests sometimes give unsatisfactorily misleading and non-predictive data for *in vivo* responses [1–3]. Recently, a number of studies have shown that 3D cell cultures provide a more physiologically relevant environment for cells and allow the study of cellular responses in a setting that resembles *in vivo* environments [2, 4–6], which suggest 3D cell culture systems may be a better option for *in vitro* studies [7].

Over the past several years, numerous 3D culture models have been investigated. Some early studies focused on the development of a variety of approaches for growing cells in 3D [8–10]. Other studies focused on the development/synthesis of various matrices for supporting cellular growth into 3D structures [11–14] and many others focused on cellular behaviors of 3D-cultured cells in comparison with 2D-cultured cells [15–19]. However, the 3D culture technology is still in its developmental stage, and better understanding and characterization/optimization/standardization of various models of the current 3D culture models are needed to more effectively utilize 3D culture in a manner suitable for high demanding applications in drug screening/discovery and cell biology studies. Some of the issues that need to be improved for current 3D models include, but are not limited to, reproducibility, cost, time-consumption, and its compatibility with many cell viability and high throughput screening assays that are currently available. With regard to reproducibility, the methods employed to form 3D spheroids and the matrix for supporting 3D growth are the two major factors that may need to be taken into account. Obviously, all 3D culture systems do not employ the same technique to form spheroids. For example, in some 3D systems, cells are grown on biological matrices, in others, cells are grown in synthetic scaffolds, or in suspension to form 3D spheroids. Variations in 3D culture systems most likely affect cellular behavior and may cause non-reproducible results among studies that employ different spheroid formation approaches. It has been noted that most published studies utilized one type of matrix to grow a particular cell line of interest in 3D culture for characterizing the cellular behaviors of the resulting 3D spheroids. As a considerable number of matrices for 3D cell cultures are available in the market, and with more being introduced frequently, more systematic evaluations of matrices for 3D culture technology are needed.

In this study, we investigated the influence of matrix on cellular behaviors by using two prostate cancer (PC) cell lines on three different matrices, and comparing cell proliferation, cellular response to anticancer drugs, and expression of drug sensitivity-associated proteins, in comparison to 2D cultured cells. The three matrices used in the study included one synthetic matrix—Alvetex, and two biologically derived matrices—the BD Matrigel™ basement membrane matrix (BD Sciences) and Cultrex® basement membrane extract (BME) (Trevigen). LNCaP, an androgen-dependent, lowly metastatic cell line, and DU145, an androgen-independent, moderately metastatic prostate cancer cell line, were used in the study [20]. We investigated how 3D matrices affect (1) the proliferative capacity of PC cells; (2) morphology of PC cell spheroids; (3) cellular response to two chemotherapeutic agents (Docetaxel and Rapamycin); and (4) the expression of proteins associated with drug sensitivity and their correlation with cellular response to the anti-cancer treatments.

## Materials and Methods

### Cell Culture

Prostate cancer cell lines, DU145 (ATCC® HTB-81™) and LNCaP (ATCC® CRL-1740™), were purchased from American Tissue Culture Collection (ATCC) (Manassas, VA, USA). Both cell lines were cultured in RPMI-1640 media (Thermo Scientific Hyclone, Logan, UT, USA) supplemented with 10% fetal bovine serum (FBS), 100 units/mL penicillin, and 0.1mg/mL streptomycin (Lonza, Walkersville, MD). All cells were propagated in standard cell culture conditions at 37°C, 5% CO_2_, and 95% humidity. Fresh media was added approximately every 2 days. Upon reaching confluency, cells were detached from flasks using 0.25% trypsin with 0.53 mM EDTA solution, followed by centrifugation to remove trypsin. The cell pellet was then resuspended in fresh media. The Vi-Cell XR cell counting system (Beckman Coulter, Miami, FL, USA) was used to determine the cell number in suspension. The desired cell densities needed for seeding in further experiments were obtained by diluting the cell suspension with fresh media.

### Preparation of 3D and 2D cultures for cell proliferation and drug response assays

Corning ® Matrigel® (growth factor reduced, phenol red free) and Cultrex® Basement Membrane Extract (BME) (growth factor reduced, phenol red free) were purchased from BD Biosciences (Bedford, MA, USA). Reinnervate® Alvetex 96-well plates and 6-well inserts were purchased from Fisher Scientific (Pittsburgh, PA, USA).

To culture cells in 3D on Matrigel or BME, 40 μL of cold Matrigel or BME was placed into the wells of a white, clear-bottom 96-well plate (Corning®) and allowed to solidify at 37°C for 45 min to form a thin gel layer. The Alvetex 96-well plate was prepared for seeding by adding 70% ethanol (100 μL) to each well, followed by two washes with complete culture media (200 μL). For 2D cell culture, due to 2D-cultured cells reaching confluency at a faster rate, cells were seeded in a 48-well plate. DU145 and LNCaP cells were seeded at an initial density of 5,000 and 7,000 cells/well in 2D and 3D matrices, respectively. Cells were left to grow for 72 or 120 h. Cell proliferation was evaluated by luminescent cell viability assay using CellTiter-Glo 3D cell viability assay (Promega Corporation, Madison, WI, USA).

For drug response tests, prostate cancer cells were grown in the various culture models as described in the section above. Cells were allowed to grow for 3 or 5 days, followed by treatment with Docetaxel (CAS 114977-28-5) or Rapamycin (CAS 53123-88-9) for 48 h. Both drugs were obtained from Cayman Chemical (Ann Arbor, MI, USA). Docetaxel and Rapamycin were dissolved in DMSO to make stock solutions and diluted with RMPI-1640 culture media to obtain the various concentrations for treatment. The stock solution for Docetaxel and Rapamycin was 1.094 mM and 15 mM, respectively.

DU145 cells were treated with Docetaxel concentrations ranging 0.01–0.2 μM and Rapamycin concentrations ranging from 0.001–0.05 μM. LNCaP cells were treated with Docetaxel and Rapamycin concentrations ranging from 0.0005–0.07 μM and 0.0005–0.01 μM, respectively. Cells without treatment and cells treated with equivalent DMSO solvent concentrations were used as controls. The final DMSO concentration in any drug treatment was less than 0.5%.

To compare the chemosensitivity of prostate cancer cells cultured in 2D and the various 3D matrices, the CellTiter-Glo® 3D cell viability assay was used to determine the cell viability after a 48 h treatment period.

### Luminescent cell viability assay (CellTiter-Glo 3D) for cell proliferation and drug response assays

#### Proliferation Assay

After the cells were grown for the desired amount of time, 100 μL of the CellTiter-Glo 3D reagent was added to each well of the 2D and 3D cultures. The plates were placed on a plate shaker for 5 min, followed by incubation for 30 min at room temperature. For 2D-cultured cells, 100 μL of the solution from each well of the 48-well plate was transferred to a white, clear-bottom 96-well plate after incubation, followed by the addition of the CellTiter-Glo 3D reagent. The luminescence was measured using the PHERAstar Plus microplate reader (BMG Labtech, Offenburg, Germany).

#### Drug Response Assay

To measure drug sensitivity to chemotherapeutics, the same protocol was followed using the CellTiter-Glo 3D assay. The luminescence readings of Docetaxel- or Rapamycin-treated cells were corrected with the readings of the vehicle control (DMSO equivalents). The cell survival percentage was normalized using the untreated cells as 100% cell survival.

### Western Blot

DU145 cells were seeded in 6-well plates at an initial cell density of 140,000 cells/well on a thin layer of Matrigel or BME (650 μL) and cultured for 3 days. One milliliter of the cell suspension was added to each well plus 2 mL of complete RPMI-1640 culture media. For 2D culture, 55,000 cells/well (1 mL) and 2 mL of media were added to each well of a 6-well plate. For Alvetex culture, 1 x 10^6^ cells in a volume of 100 μL was placed onto a prepared 6-well insert, which was placed into the well of 6-well plate. The 6-well plates containing the Alvetex inserts were incubated for 45 minutes to facilitate cell attachment to the scaffold. Supplemented culture media (7 mL) was then added to each well. The same steps were followed for LNCaP cells. LNCaP cells were seeded at a density of 230,000 cells/well in Matrigel and BME; 70,000 cells/well in 2D culture; and 1.5 x 10^6^ cells/insert in the Alvetex matrix. Cells were then grown for 3 days before lysates were prepared.

Proteins were extracted using M-PER lysis buffer (Thermo Scientific). M-PER was supplemented with protease and phosphatase inhibitors, sodium fluoride, and PMSF. For 3D cultures, Matrigel and BME were liquefied on ice using the Cell Recovery Solution (BD Biosciences, Bedford, MA, USA) before adding the lysis buffer. The disc of the Alvetex insert was removed from the insert clip and placed into a microcentrifuge tube containing supplemented M-PER lysis buffer. For 1 h, the tube was vortexed every 5 min for 10 s in order to facilitate partial protein recovery. The soluble protein concentration was determined using the Pierce™ BCA protein assay kit (Thermo Scientific).

Proteins (~20 μg per well) were loaded onto and separated on a NuPAGE Bis-Tris mini gel (Invitrogen) with MOPS buffer and transferred to an Immobilon-FL PVDF membrane (EMD Millipore) using a semi-dry transfer unit. Membranes were blocked for 1 h at room temperature with a 1:1 LI-COR blocking buffer-TBS solution. Membranes were probed overnight at 4°C with primary antibodies against the protein of interest and a loading control for normalization. For DU145 cells, total EGFR and β-III tubulin protein expression was examined using monoclonal anti-EGF Receptor (Cell Signaling, #4267) and monoclonal anti-TUBBIII (Covance Inc, #MMS-435P) antibodies at a 1:1000 dilution. GAPDH and β-actin were used as loading controls. The monoclonal anti-GAPDH antibody (Thermo Scientific, MA5-15738) and polyclonal anti-β-actin antibody (Thermo Scientific, PA5-16914) were used at a 1:1500 dilution. In LNCaP cells, p53 expression was examined using the polyclonal anti-p53 (Cell Signaling, #9282) at a 1:1000 dilution. β-actin was used as the loading control. The monoclonal anti β-actin antibody (Thermo Scientific, MA5-15452) at a 1:2000 dilution was used. The secondary antibodies (1:5000), goat anti-mouse and goat anti-rabbit labeled with IR 680 and IR 800 were all obtained from LI-COR (Lincoln, NE, USA). Membranes were incubated at room temperature for 1 h with secondary antibodies, followed by 5 washes with TBS-T (0.1% Tween) for 5 min. Blots were then imaged using the LI-COR Odyssey imaging system. To account for potential variations in protein loading, the relative fluorescence units (RFU) of bands for each protein of interest were normalized to the RFU values of the loading controls.

### Microscopic Imaging

Optical microscopic images of 2D and 3D cultures (Matrigel and BME) were taken using the Nikon ECLIPSE E600FN microscope (Roper Scientific, Inc. Photometric, Tucson, AZ, USA). Fluorescent images of cells in all 3D matrices were taken using the BD Pathway 855 Bioimager (BD Biosciences, San Jose, CA, USA). For fluorescent imaging, prostate cancer cells were stained with blue Hoechst 33342 and CellTracker Red CMTPX dye (Invitrogen, Carlsbad, CA, USA).

Briefly, cells were grown in Matrigel or BME on 8-chamber glass slides or on Alvetex 6-well inserts for 3 days. Each chamber was coated with ~90 μL of Matrigel or BME and incubated before seeding cells. Alvetex inserts were prepared according to the manufacturer's guidelines prior to cell seeding. Following 3 days of growth, cells were stained with Hoechst 33342 (10 g/mL) and CellTracker Red (10 μM) in 100 μL of fresh base media for 30 min. Cells were then washed with PBS twice, followed by fixation with 2% formaldehyde in PBS for 10 min at room temperature. Cells were washed again with PBS, and ProLong Gold Antifade Reagent (Cell Signaling) was added to each chamber or to the Alvetex insert. The chamber slide and glass slide with the Alvetex disc were then incubated for 2 h. The slides were placed directly onto the stage of the BD Pathway for fluorescent imaging.

### Statistical Analysis

All statistical analyses in this study were performed using one-way Analysis of Variance (ANOVA) and unpaired t-tests. p≤0.05 was considered as a significant difference.

## Results and Discussion

### Effect of Matrices on the Proliferation of 3D-Cultured Prostate Cancer Cells

**Fig 1:**
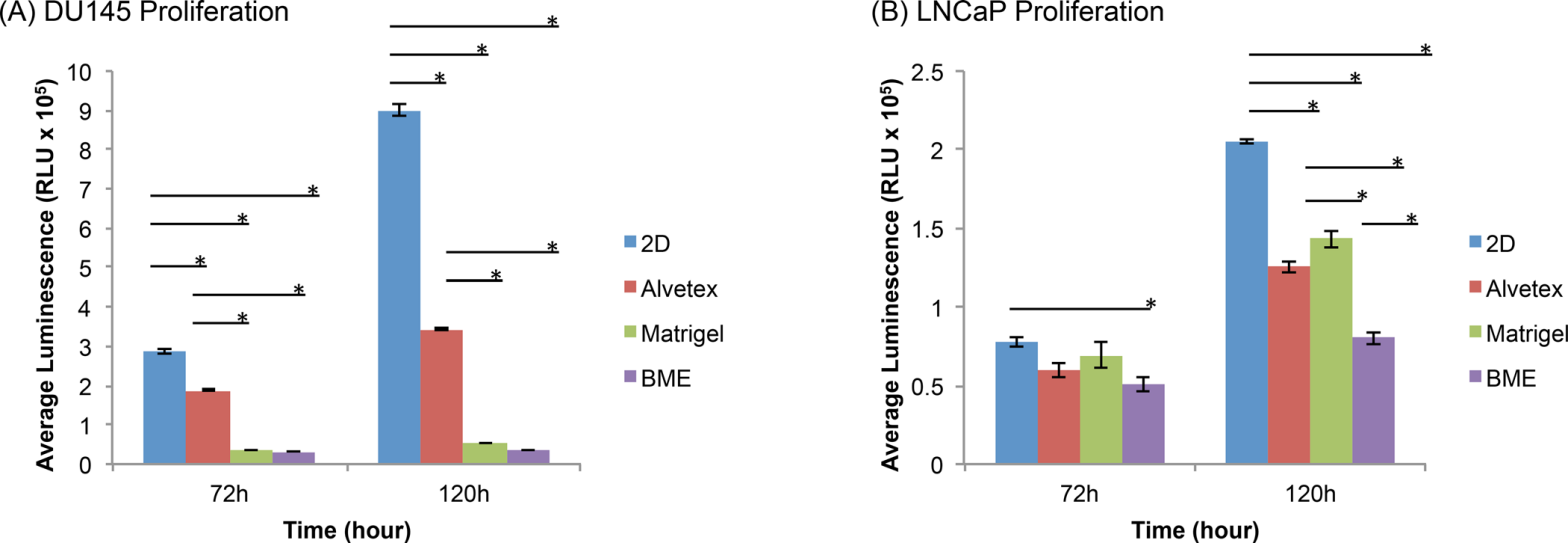
Prostate cancer proliferation of 3D- and 2D-cultured cells. (A) DU145 and (B) LNCaP cells were grown in 3D matrices (Alvetex, Matrigel, and BME) and 2D culture. Cell viability measured at 72 and 120 h of growth. Initial cell density was 5,000 cells per well. Results obtained using CellTiter-Glo 3D cell viability assay. Asterisks represent a significant difference between groups. *p≤0.05 using one-way ANOVA and unpaired t-tests.

**Fig 2:**
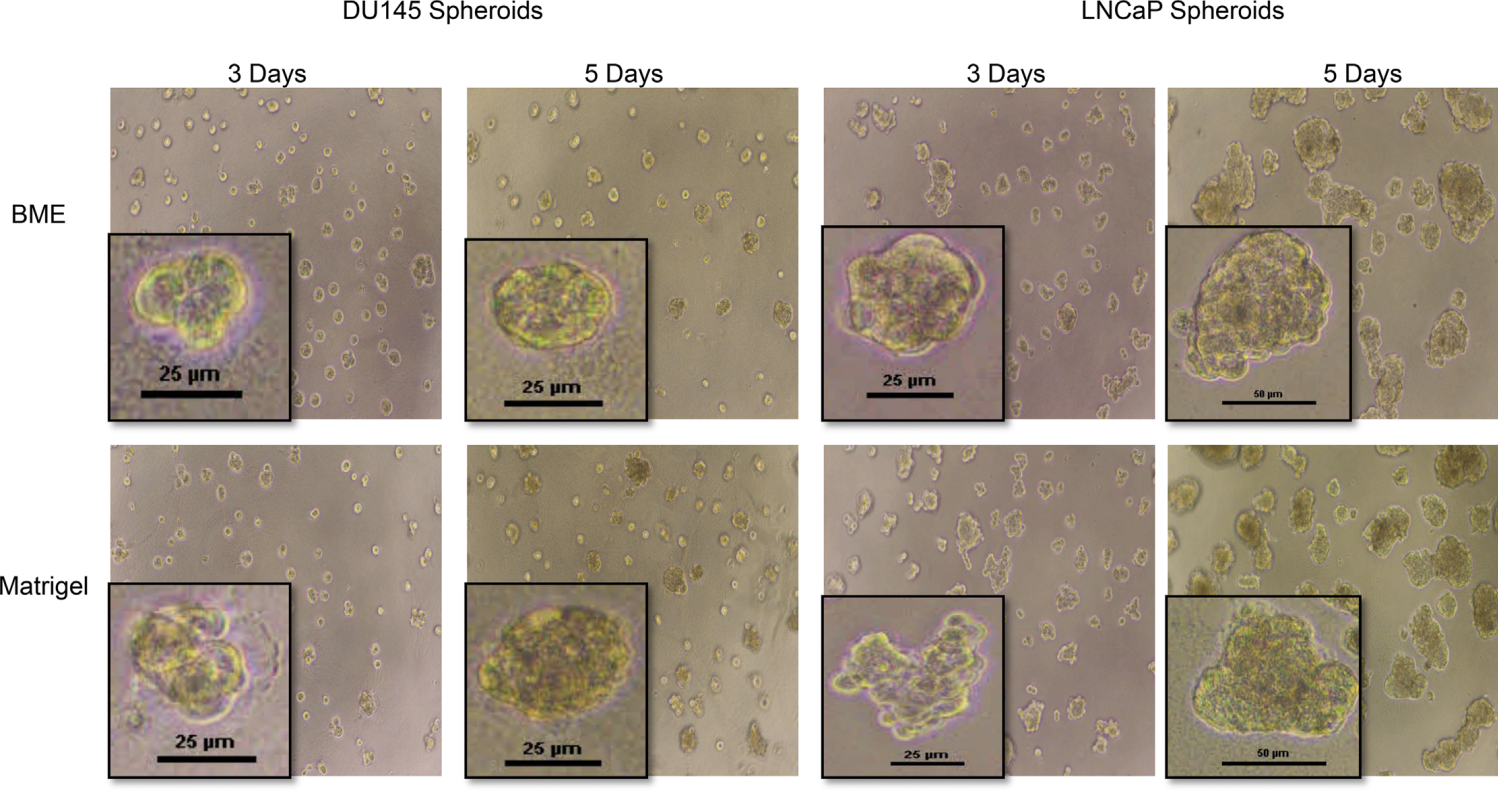
Optical microscopic images of DU145 and LNCaP spheroids in Matrigel and BME after 3 day and 5 day growth period. (A) DU145 and (B) LNCaP cells were seeded onto Matrigel- and BME- coated wells and grown for 3 or 5 days. Scale bars indicate the width of spheroid, and are shown at 25 and 50 μm.

PC cell lines, DU145 and LNCaP, were grown on three different matrices—Matrigel, BME, and Alvetex. Cell proliferation rates at 72 and 120 h were determined by measuring cell viability using the CellTiter Glo 3D assay kit. Fig 1 shows the comparison of cell proliferation rates of DU145 cells (Fig 1A) and LNCaP cells (Fig 1B) on the three types of matrices, along with their counterpart 2D-cultured cells. As shown in Fig 1A, 3D-cultured DU145 cells on all three matrices proliferated slower than in traditional 2D culture at each time point. Among the 3D-cultured cells, Alvetex-cultured DU145 cells proliferated at the highest rate, followed by those in the two biological matrices, Matrigel and BME, which had similar proliferation rates. After 72 h, the average luminescence, which indicates the presence of viable cells, increased in Alvetex- and 2D-cultured cells, showing that cells continued to proliferate; however, there was no significant difference in the proliferation of DU145 cells cultured on the two biological matrices, Matrigel and BME, between 72–120 h (Fig 1A). Fig 2A also shows that the spheroid size of DU145 spheroids on Matrigel and BME did not change between 72 and 120 h. At both time points, the spheroid size was ~25 μm.

For LNCaP cells (Figs 1B and 2B), at 72 h, the cells proliferated at fairly similar rates in all 3D models and in 2D culture. However, at 120 h, a significant difference in the relative number of viable cells appeared between cells cultured in the various culture systems. 3D spheroids of LNCaP cells cultured on the three matrices exhibited a significantly slower proliferation rate than cells cultured in 2D. Among the 3D spheroids, cells cultured on Matrigel exhibited the fastest proliferation rate, followed by LNCaP cells in Alvetex, and lastly, cells on BME.

The results indicated that both 3D-cultured DU145 and LNCaP cells in general proliferated at a slower rate compared to those cultured in the traditional 2D cell culture. Such observations are in agreement with many studies showing that various cell lines proliferate at a reduced rate in 3D cultures compared to those cultured in 2D [15–19, 21, 22]. Although some cell lines have been shown to proliferate faster in 3D cultures than 2D culture [23], it is more common for cells to proliferate slower in 3D culture systems. Whether or not the proliferation rate of 3D-cultured cells is slower or faster relative to its counterpart in 2D, the proliferation characteristics of 3D-cultured cells typically better represent the growth of tumor cells *in vivo* [21]. The increased proliferation rates of DU145 and LNCaP cells under 2D conditions is likely due to the monolayer structure, in which more cells are exposed to the nutrients and growth factors of culture medium, making them more likely to proliferate at a fairly uniform rate across the flat surface. Other factors contributing to differences in 2D- and 3D-cultured proliferation rates are cell-cell and cell-ECM interactions that may alter the expression of genes and proteins associated with cellular growth and proliferation [21, 24, 25]. Nevertheless, it is clear that the proliferation rates of cells cultured in 3D and 2D are usually different and are cell line dependent [26]. It was also noted that the difference in proliferation in 3D and 2D became more pronounced as the culture time increased, as observed in both DU145 and LNCaP cell lines (Fig 1).

With regard to the proliferation rate of cells cultured in 3D on different matrices, the results showed that the proliferation rate of DU145 cells differed between the various 3D systems (Fig 1A). There was a significant difference between the proliferative capacity of Alvetex-cultured cells and those in Matrigel and BME in which DU145 3D spheroids exhibited similar proliferation rates. This observation suggests that the difference in cell proliferation could be due to the different nature of matrices. Alvetex is a synthetic, highly porous polystyrene scaffold. Cells are seeded onto the porous scaffold and migrate into the pores over time, where they aggregate to form spheroid structures [27]. Matrigel (BD Biosciences®) and Cultrex BME (Trevigen ®) are both biologically-derived matrices. They are reconstituted basement membrane extracts derived from the Engelbreth-Holm-Swarm (EHS) mouse sarcoma, and are rich in ECM constituents including laminin, collagen IV, perlecan, nodigen/entactin, proteases, as well as growth factors including epidermal growth factor (EGF) and insulin-like growth factor (IGF)[28, 29]. It is noted that there is a difference in protein concentration between Matrigel and Cultrex BME, with typically 8–12 mg/mL in Matrigel [30] and 15–17 mg/mL in BME, respectively. To our knowledge, there are no current studies that have compared the use of these matrices for 3D spheroids growth, despite there being a few studies that have noted the role of matrix in 3D cell growth. For example, it has been reported that rat embryonic stem cells (RESCs) grown in a synthetic poly-(L-lactic acid) (PLLA) scaffold proliferated at a faster rate compared to RESCs in BME [29]. Synthetic scaffolds support spheroid formation by providing a constructed porous structure for cells to grow into 3D structures inside the pores. But because of the lack of proteins and growth factors in synthetic scaffolds, cellular proliferation in synthetic/artificial matrices is often more similar to that of 2D-cultured cells. On the other hand, biological matrices support the growth of 3D spheroids by providing an environment with proteins and growth factors that closely mimic the natural environment of cells *in vivo*. 3D spheroids in Matrigel and BME exhibited proliferation rates resembling those *in vivo*, which were much slower than 2D-cultured cells. Another factor that may contribute to the differences in cell proliferation on various matrices is the matrix stiffness. It was noted that the influence of matrix on LNCaP proliferation in 3D spheroids was not as pronounced as what was seen in DU145 cells. As shown in Fig 1B, at 72 h, the proliferation rate of LNCaP cells was very close between cells in all 3D systems. Differences in proliferative capacities of LNCaP cells cultured in different models did however appear at 120 h. Interestingly, the difference between the synthetic matrix and biologically derived matrices was not seen in LNCaP 3D cultures at all time points, whereas the difference between the two biologically derived matrices, Matrigel and BME, was evident. The difference observed in the proliferative capacity of cells in Matrigel and BME at 120 h (Fig 1B) may be due to differences in protein concentrations and matrix stiffness between the two biological matrices. Studies have shown that a higher protein concentration correlates with a higher matrix stiffness; therefore, it is plausible that BME, which typically has a higher protein concentration compared to Matrigel, is a stiffer matrix. A study by Sieh et al. showed that matrix stiffness influenced LNCaP proliferation in polyethylene glycol (PEG) hydrogels, in which it was observed that the proliferation of LNCaP cells decreased as matrix stiffness increased [31]. Our results on LNCaP cell proliferation on undiluted, 1:1, and 1:2 diluted Matrigel and BME for 72 h were consistent with the reported study [31] in that increased cell proliferation rate was observed on diluted Matrigel or BME (lower protein concentrations) (S1 Fig). However, diluted Matrigel and BME did not consistently support spheroid formation; therefore, they were not selected for further cell proliferation or drug response tests in this study.

A further comparison between the proliferation of two cell lines showed that LNCaP cells exhibited an increase in the relative number of viable cells from 72 to 120 h, while DU145 cells showed little or no increase in cell growth between 72–120 h in the 3D systems (Fig 1 and Fig 2). These observations suggested that the nature of a cell line also play a role in the proliferation rate on these matrices. LNCaP cells are androgen-dependent PC cells that secrete prostate specific antigen (PSA), whereas DU145 cells do not secrete PSA. PSA is a serine protease that has been shown to degrade ECM glycoproteins, fibronectin, and laminin. It has been reported that deregulation and degradation of the ECM or basement membrane may increase proliferation by modulating the activity of growth factors and growth factor receptors [32–34]. However, further tests on another prostate cancer cell line–PC3 cell line, which is a androgen-independent and non-PSA secreting cell line, on Matrgel and BME showed that its growth pattern between 72 h to 120 h was similar to LNCaP cells (S2 Fig), which suggested that the secretion of PSA was not necessarily associated with the increased proliferation of LNCaP 3D spheroids among the biological matrices between 72 and 120 h (Fig 1B). There are likely many other factors that play a role in the proliferation rate of PC cells in various 3D models, for example, androgen receptor status, expression of matrix metalloproteinases[35, 36], metastatic characteristics, the growth factors that are able to bind to cell surface receptors to promote cellular growth [37]. Nevertheless, the results clearly indicated that the proliferation of prostate cancer cells in 3D cultures was cell-line dependent.

Although all both the DU145 and LNCaP cell lines proliferated fastest in 2D culture, their proliferative capacity in 3D matrices differed. It was noted that after 120 h of cellular growth, DU145 cells proliferated at the fastest rate in Alvetex, while LNCaP cells exhibited the highest proliferative capacity in Matrigel. Collectively, these findings showed that not only was prostate cancer proliferation influenced by 3D matrix type, but also by the characteristics of each cell line.

### Spheroid Structures Formed on Matrices

It is reported that different cell types may form 3D spheroids with distinct structures in various 3D models. The spheroid structures can be classified into different types depending on their morphological appearance, structural arrangement of cells, and the nature of cell-cell interactions. Kenny et al. [38] classified the structures of 3D spheroids formed by a panel of 25 breast cancer cell lines into four groups: round, mass, grape-like, and stellate. Harma et al. [39] also reported 3D spheroid structures for a comprehensive panel of prostate cancer cell lines, and found that most structures fell into the 4 categories with the exception of a few cell lines that failed to form spheroids. The structural characteristics and the nature of cell-cell interactions in the four spheroid types have been described in detail previously [16, 26, 38–41]. The structure of 3D spheroids have been shown to greatly influence drug efficacy/response. Cell-cycle distribution, intracellular contact, the microenvironment within the spheroid, and drug penetration are also factors believed to influence the cellular response to anti-cancer treatments [41].

**Fig 3:**
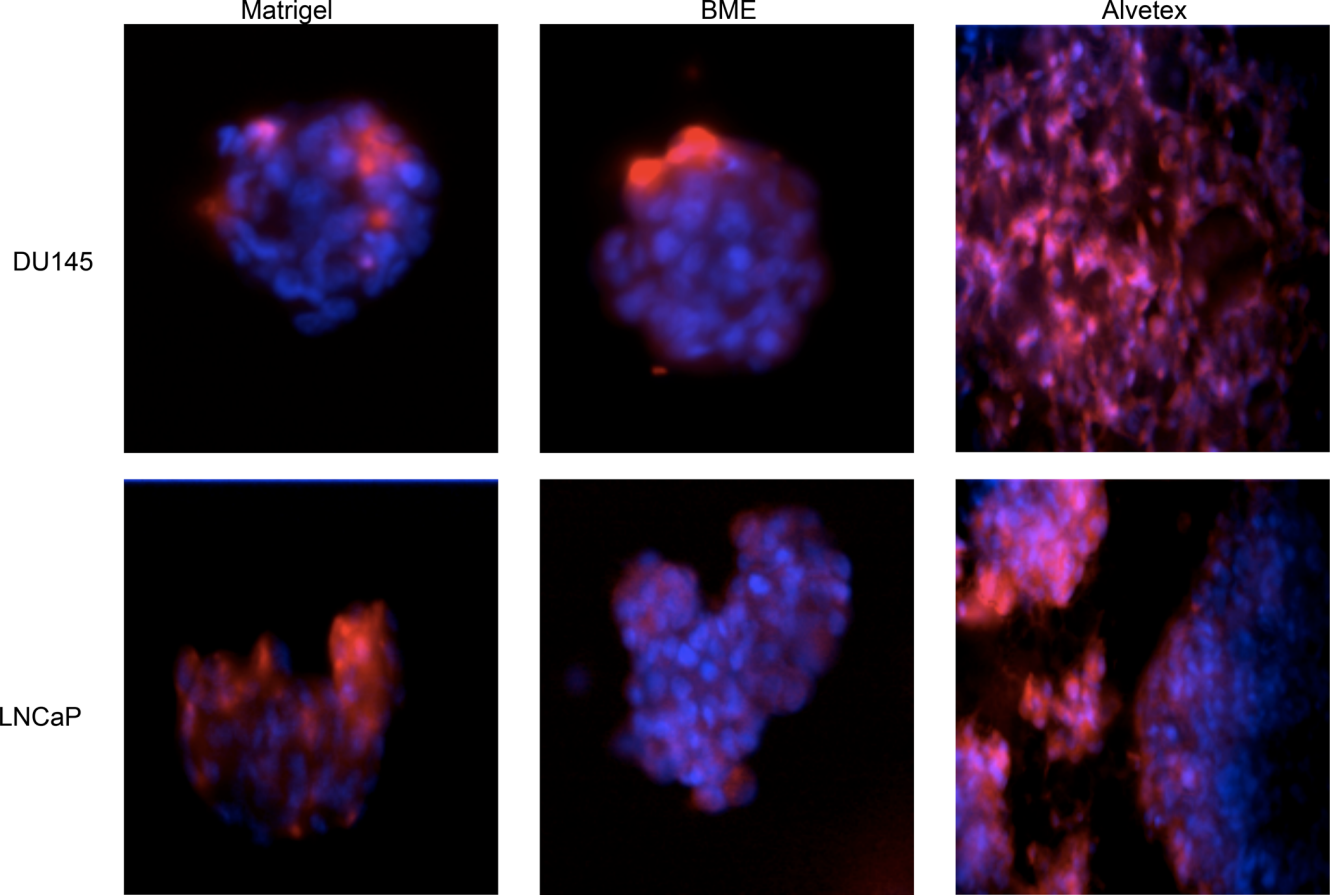
Fluorescent microscopic images of DU145 and LNCaP cells cultured in the 3D matrices: Matrigel, BME, and Alvetex. Cells were seeded onto the various matrices and cultured for four days. Hoechst 33342 (blue) and CellTracker Red CMPTX dye (red) was used to stain nuclei and cytoplasm, respectively. Images obtained using BD Pathway Bioimager.

Fig 3 shows the fluorescent images of the 3D spheroids of DU145 cells and LNCaP cells cultured in the three matrices. Based on the characteristics of the spheroids, DU145 spheroids on both Matrigel and BME can be classified as round-type spheroids (Fig 3). LNCaP cells cultured in Matrigel and BME formed larger, irregular spheroids that did not exhibit the same roundness as DU145 spheroids, but instead exhibited the mass phenotype (Figs 2 and 3). In Alvetex, DU145 cells in Alvetex seemed to adhere, accumulate, and grow on top of the porous matrix, forming a thick layer of cells with no defined spheroid structure (Fig 3). On the other hand, LNCaP cells formed spheroid structures in Alvetex, which exhibited the mass-type spheroid as seen in Matrigel and BME (Fig 3). The results indicated that cells of the same cell line (DU145 vs LNCaP) formed the same spheroid type on similar biological matrices (Matrigel and BME), which suggested that the spheroid type formed on 3D matrices is primarily cell line dependent. On the synthetic Alvetex matrix, it was also a cell line dependent situation. DU145 cell line failed to form a defined 3D structure, while LNCaP cell line formed well defined 3D spheroid structures (LNCaP) in the same way as on biological matrices. Between LNCaP and DU145 cells, the latter cell line is more adherent, which may account for differences in spheroid formation on Alvetex. The adherent nature of DU145 cells may cause their inability to fully penetrate into the pores of the matrix and form spheroid structures.

### Cellular Response of 3D-Cultured Prostate Cancer Cells to Chemotherapeutics

We further investigated the cellular responses of the 3D cultured DU145 and LNCaP cells on the different matrices, aiming to determine whether the matrix type influences the cellular drug response, and furthermore, if the cellular response is correlated to the matrix's influence on cell proliferation and/or spheroid structures. 3D- and 2D-cultured DU145 and LNCaP PC cells were treated with the two anti-cancer drugs: Docetaxel (DOC) and Rapamycin (RAP). Docetaxel is a microtubule stabilizing taxane that binds to β-tubulin in assembled tubulin, reducing depolymerization. This prevents the normal formation of mitotic spindles, leading to mitotic arrest, and eventual cell death [42, 43]. Rapamycin, also known as Sirolimus, is a macrocyclic triene antibiotic that exhibits antifungal, immunosuppressive, and anticancer properties. The drug inhibits cancer cell growth by binding to the mammalian Target of Rapamycin (mTOR). mTOR and its downstream effectors play an important role in the promotion of cell growth, proliferation, and cell survival; therefore, Rapamycin inhibition of mTOR leads to decreased proliferation and survival of tumor cells [44–46].

#### Response of DU145 3D Spheroids to Chemotherapeutics

**Fig 4:**
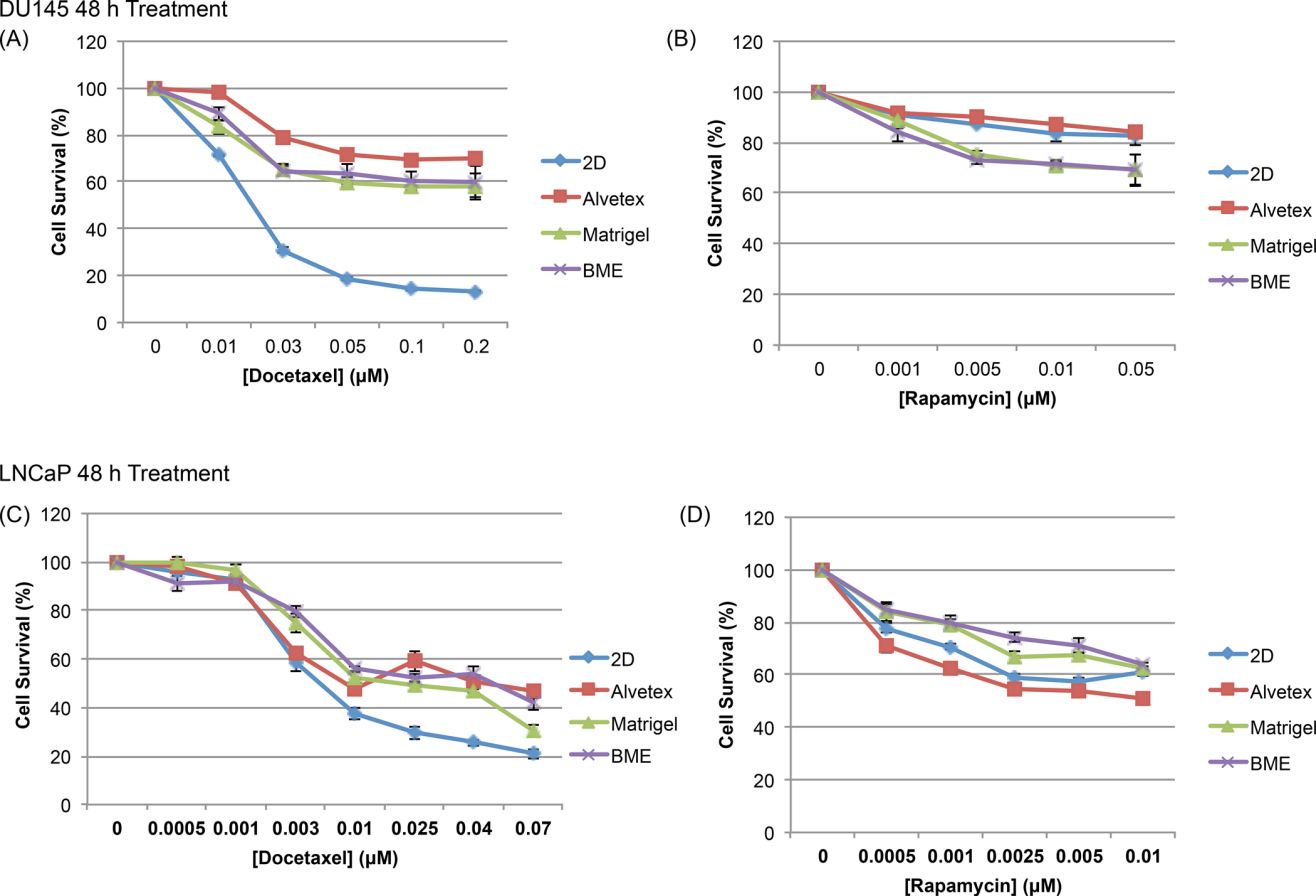
Prostate cancer cell survival after treatment with chemotherapeutics. (A and B) DU145 and (C and D) LNCaP cells in 2D and 3D matrices were exposed to various concentrations of Docetaxel and Rapamycin for 48 h. Cell survival is calculated as a percentage of the ratio between treated and untreated cells (Cell Survival %). Data is expressed as mean ± SEM based on at least three independent experiments with triplicate wells for each drug concentration.

Fig 4A shows the cell survival percentage of DU145 cells cultured in 2D and 3D in response to a range of concentrations of DOC and RAP, at 48 h post treatment. Based on the cell survival percentage, 3D-cultured cells on all three matrices were more resistant to Docetaxel treatment in comparison to cells cultured in 2D. Being that DOC is an anti-mitotic drug, it is more likely to target rapidly dividing/proliferating cells. DU145 cells proliferate in 3D culture on all three matrices at a significantly slower rate than those in 2D system, so the reduced sensitivity of 3D-cultured cells was correlated with their slower proliferative capacity in comparison to 2D-cultured cells (Figs 1A and 2A). Among the 3D matrices, cells in Matrigel and BME responded similarly to DOC treatment, which was expected since they had similar proliferation rates and formed the same type spheroid structure on the two biological matrices. However, it was surprising that cells cultured in Alvetex were slightly more resistant to DOC treatment compared to those on two biological matrices, although the proliferation rate of Alvetex-cultured DU145 cells proliferated significantly faster than those on the two biological matrices. This suggested that the cellular response of cells on Alvetex to DOC was not merely correlated to proliferation rates, but could be due to the synthetic nature of Alvetex scaffold, as Alvetex is not as biologically relevant, lacking ECM components involved in various signaling pathways that play a role in cell behavior including their response to treatment [21]. In addition, DU145 cells failed to form typical 3D spheroids in Alvetex. The structure formed in Alvetex obviously differed from the 3D spheroid structure of those in Matrigel and BME (Fig 3), which may affect cell-cell communication and/or drug penetration into the cell culture, thereby causing cells in the synthetic and biological matrices to respond to DOC treatment differently.

In response to Rapamycin treatment, a different pattern was observed in comparison to DOC treatment. 3D spheroids of DU145 cells cultured on Matrigel and BME were more sensitive to RAP than those cells cultured in 2D; and cells cultured in the Alvetex matrix exhibited a response similar to 2D-cultured cells. It seemed DU145 cellular response to RAP was more likely associated with the culture structure formed on biological vs. synthetic matrix. While 3D spheroids on Matrigel and BME were supported by ECM components and formed similar round type spheroids, alvetex-cultured and 2D cultured cells both lacked ECM support in cell growth and did not form 3D structure. In Alvetex, DU145 cells form an undefined structure in Alvetex (Fig 3), in which cells tended to attach to the matrix surface and failed to fully penetrate into the porous scaffold. This would mean that DU145 cells in Alvetex had similar exposure to culture medium as 2D cultured cells, causing the similar response to RAP treatment of Alvetex-and 2D- cultured DU145 cells.

Overall, the results indicated that matrix type influenced the cellular response of 3D cultures to drug treatments, but the underlying mechanisms were very complex, involving the influence of matrices on the proliferation rate of cells, the spheroid structure, and fine cell-cell and cell-ECM interactions. In view of the results on the same cell line but to different drugs, it was also clear that a drug's mechanism of action definitely played a role in the way and to the extent in which the matrix type influenced the cellular response to a drug.

#### Response of LNCaP 3D Spheroids to Chemotherapeutics

Fig 4B shows that the influence of matrix on the cellular response of 3D-cultured LNCaP cells to DOC exhibited a similar pattern as DU145 cells, in that all 3D-cultured cells were more resistant to DOC than 2D-cultured cells. Upon Docetaxel treatment, the viability of 3D-cultured LNCaP cells was significantly higher compared to those in 2D culture at higher concentrations beyond 0.01 μM. This finding is in agreement with a previous study which showed that 3D-cultured LNCaP cells in a high-throughput culture platform were more resistant to Docetaxel treatment in comparison to 2D-cultured cells [47]. At lower concentrations, there was little difference in the drug response between cells in the various cultures (Fig 4B).

Although not significant at every concentration, 3D-cultured cells on Matrigel and BME were generally more resistant to RAP treatment than 2D-cultured cells, and Alvetex-cultured cells behaved similarly to cells in 2D (Fig 4D). Amongst the three matrices, the results highlighted a key difference between biological matrices and the synthetic matrix. The resistance of Matrigel- and BME-cultured cells to RAP could be due to the gelatinous nature of these biological matrices, making it more difficult for drugs such as Rapamycin to reach their target, as previous studies have suggested that biological matrices may also prevent drugs from reaching the spheroid, thereby decreasing sensitivity to treatment [48–50].

#### Effect of Spheroid Size on Drug Response and the Relative Trend in Resistance

**Fig 5:**
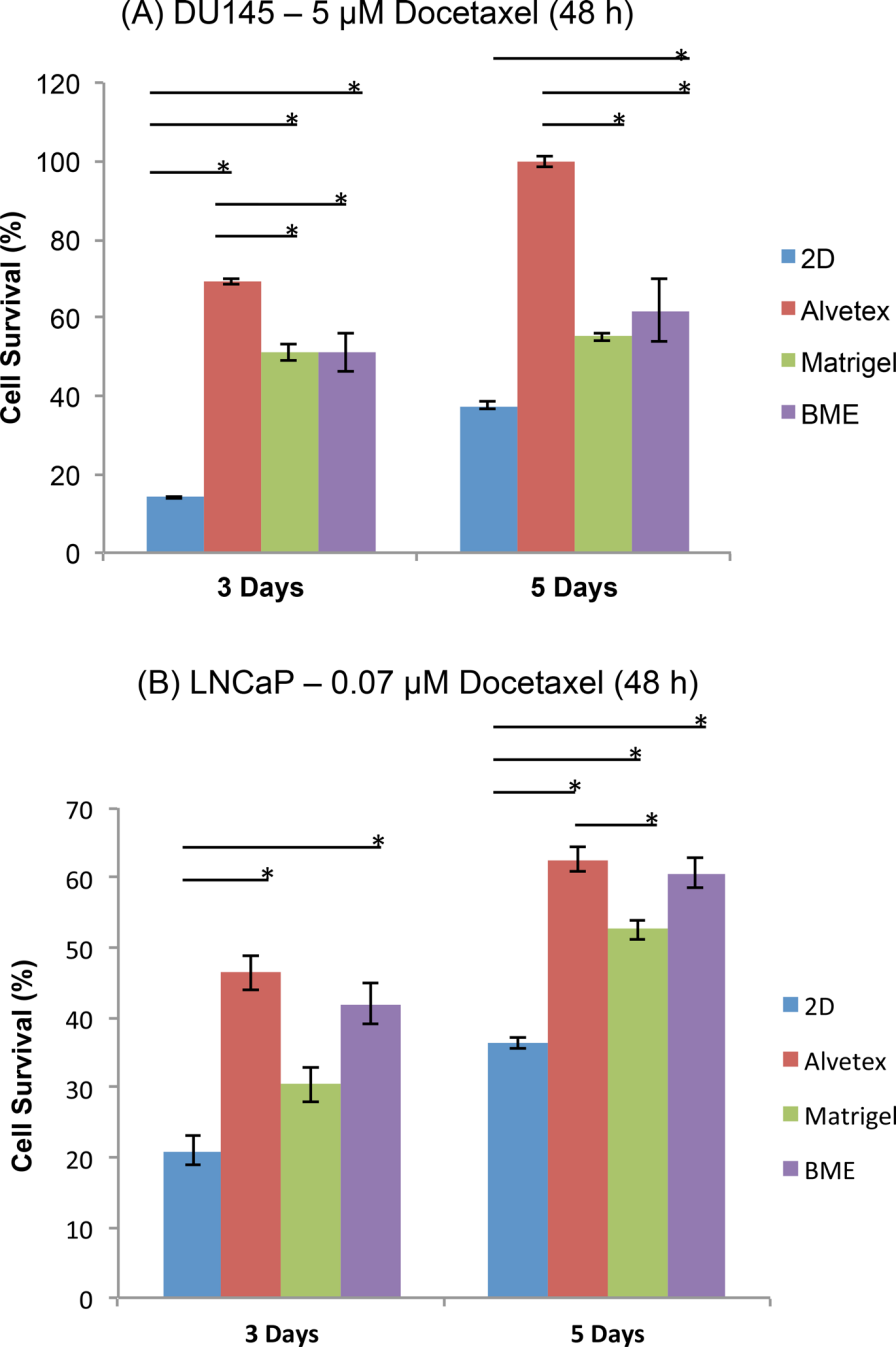
Survival of 3 Day and 5 Day spheroids after Docetaxel treatment. (A) DU145 and (B) LNCaP cells were grown for 3 or 5 days, followed by Docetaxel treatment for 48h at a concentration of 5 μM and 0.07 μM, respectively. Cell survival was calculated as a percentage of the ratio between treated and untreated cells (Cell Survival %). Data is expressed as mean ± SEM based on at least three independent experiments with triplicate wells for each drug concentration.

DU145 and LNCaP cells were cultured on the three types of matrices for 3 or 5 days, followed by treatment with Docetaxel for 48 hours. Fig 5 shows the 3-day and 5-day spheroids of DU145 cells (Fig 5A) and LNCaP cells (Fig 5B) in response to Docetaxel. For both cell lines, as the size of spheroids increased, cellular resistance to Docetaxel treatment increased, shown by the increased cell survival on each type of matrix when the spheroid size increased from 3-day to 5-day. This is consistent with the observations in previous studies which have shown that larger spheroids often exhibit increased resistance to treatment [51, 52]. However, it is also important to note that the relative sensitivity (the trends in resistance) to DOC treatment remained consistent between 3-day and 5-day cultured cells on the three matrices and 2D-cultured cells. This observation suggested that the influence of matrix type on the drug response of PC cells remain unchanged over time, which is important when it comes to 3D matrix selection for future studies, particularly those in drug screening and discovery.

### Expression of Drug Sensitivity-Associated Proteins in 3D Spheroids Cultured on Different Matrices

To look further into the influence of matrix type on the cellular response of 3D spheroids to anti-cancer treatments, we examined the expression of drug sensitivity-associated proteins in 3D spheroids of DU145 and LNCaP cells cultured on the three matrices. Literature has shown that the protein expression profile of 3D-cultured cells often differs from their 2D-cultured counterparts. Proteins, including those associated with a drug's mechanism of action, sensitivity, or resistance are often differentially expressed between 2D monolayer culture and 3D cultures [21, 53–55], but little is done to investigate whether the expression of drug sensitivity-associated proteins differs between 3D spheroids cultured on various matrices.

#### Drug-Associated Protein Expression in DU145 Cells

Epidermal growth factor receptor (EGFR) is a cell surface receptor tyrosine kinase, whose activation stimulates the activation of downstream signaling pathways including the PI3K/AKT/mTOR pathway which is involved in cell growth, proliferation, and survival [56]. Its over-expression has been reported in many cancer types. Although the expression of EGFR has not been directly associated with Rapamycin sensitivity, studies have shown that EGFR inhibitors often increase the sensitivity of cancer cells to Rapamycin and vice versa, Rapamycin often enhances the effectiveness of EGFR inhibitors [57–60]. Therefore, we expected that increased expression of EGFR would reduce the sensitivity of DU145 cells to Rapamycin. β-III tubulin is a component of microtubules, which are composed primarily of α- and β-tubulin heterodimers, and plays a critical role in cell division regulation. There are several β-tubulin isoforms and each isoform is expressed in a tissue-specific manner. Under normal conditions, β-III tubulin is expressed at low levels in most cells besides neurons and sertoli cells of the testis [61]. However, cancer cells (i.e. prostate, lung, pancreatic, and breast) often exhibit increased expression of β-III tubulin, which has been associated with chemoresistance as well as increased cell survival [61–63]. Although the role of β-III tubulin has not been well characterized in prostate cancer, studies have shown that over-expression of this protein in several other cancers (i.e. breast and ovarian cancer) is associated with resistance to taxane-based chemotherapeutics such as Docetaxel [44, 63–65]. In this study, we examined the expression of EGFR and β-III tubulin in 3D- and 2D-cultured DU145 cells in an attempt to see if the expression of these proteins was influenced by the matrix type, and whether their expression was correlated with the cellular response to Rapamycin and Docetaxel.

**Fig 6:**
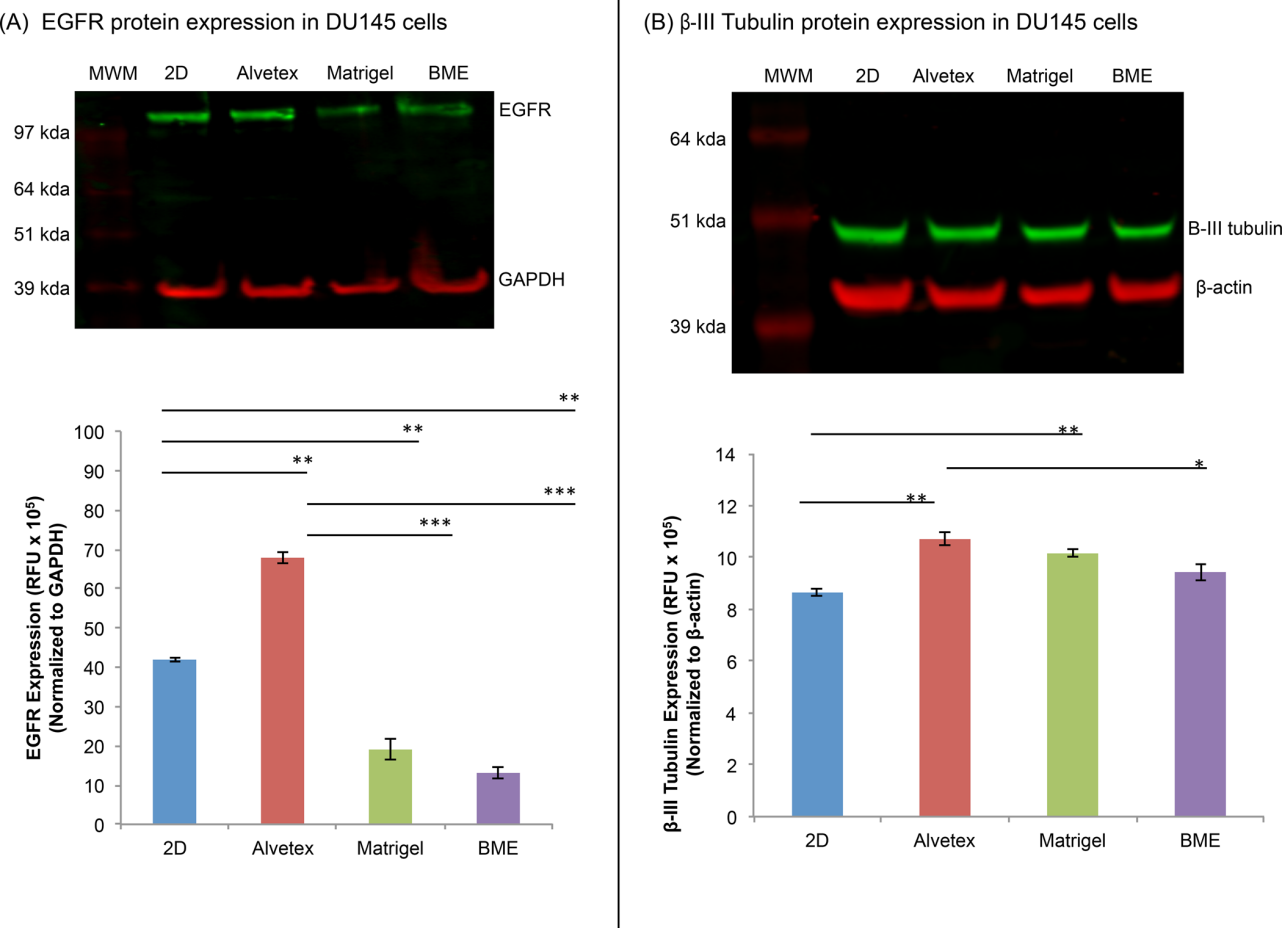
Total EGFR and β-III tubulin protein expression in DU145 cells. A representative image of the expression of (A) total EGFR and (B) β-III tubulin in 2D- and 3D-cultured DU145 cells. Bars represent the relative protein quantification of (A) total EGFR and (B) β-III tubulin on the basis of GAPDH and β-actin, and indicate the mean ± SEM (n = 3 for each group). *p≤0.05, **p≤0.01, and ***p≤0.001, using one-way ANOVA and unpaired t-tests.

Fig 6 shows the expression of EGFR (Fig 6A) and β-III tubulin (Fig 6B) in DU145 3D spheroids cultured on three matrices, along with 2D-cultured cells. Among the 3D-cultured cells, the expression of EGFR in Alvetex-cultured cells was significantly higher compared to those on the two biological matrices, with 5.2x and 3.6x higher than those in Matrigel and BME, respectively. It was also significantly higher than that in 2D-cultured cells (Fig 6A). Referring back to the dose response curve for Rapamycin-treated DU145 cells (Fig 4B), there was an inverse correlation between EGFR expression and Rapamycin sensitivity of the cells cultured on the different matrices. This is consistent with previous reports which have shown that over-expression of the EGFR protein has been associated with chemoresistance in several types of cancer [66–68].

EGFR over-expression has been implicated in prostate, esophageal, breast, ovarian, colorectal, head and neck, and non-small cell lung cancer [67–70]. Weihua et al. reported that EGFR, independent of its kinase activity, contributes to the survival of tumor cells by maintaining basal intracellular glucose levels, thereby preventing cells from starving and undergoing autophagy. This is believed to increase the ability of cancer cells to survive even upon treatment with anti-cancer drugs and tyrosine kinase inhibitors [71]. DU145 cells in Alvetex and 2D culture expressed higher levels of EGFR compared to those in Matrigel and BME, likely due to the artificial versus biological nature of the matrices. Compared to 3D spheroids on Matrigel and BME, cells in Alvetex and 2D culture were more likely to be exposed to the culture medium and its growth factors. The growth factors can then bind to and stimulate EGFR expression in 2D- and Alvetex-cultured DU145 cells, which caused them to have a higher percentage of viable cells upon RAP treatment in comparison to cells in the biological matrices, Matrigel and BME [71, 72].

Fig 6B shows that cells cultured in all 3D matrices expressed higher levels of β-III tubulin in comparison to their 2D-cultured counterparts. The expression of β-III tubulin in 3D-cultured DU145 cells followed a decreasing trend from Alvetex-, Matrigel-, and BME-cultured cells. Referring back to the DU145 Docetaxel response curve (Fig 4A), cells in the Alvetex matrix were more resistant to Docetaxel treatment, followed by cells in Matrigel and BME, and lastly, 2D. Therefore, our results showed an inverse correlation between the resistance of DU145 cells to Docetaxel and the expression of β-III tubulin. These results are consistent with a previous observation which showed that increased expression of β-III tubulin was associated with reduced sensitivity to taxane-based therapies [63]. These results suggested that differences in matrix type may induce differential expression of proteins in 3D-cultured DU145 cells, which in part, may lead to differences in the cellular response to Rapamycin and Docetaxel treatment.

#### Drug-Associated Protein Expression in LNCaP Cells

In LNCaP prostate cancer cell line, the protein expression levels of p53, which has been reported to be associated with Docetaxel sensitivity, was examined. The tumor suppressor p53 is referred to as the "guardian of the genome" because of its ability to recognize DNA damage and induce cell cycle arrest or apoptotic cell death[73, 74]. LNCaP cells express wild-type p53, which functions as a pro-apoptotic protein in response to DNA damage [75]. Liu et al. [76] showed that p53 status determined how prostate cancer cells responded to Docetaxel treatment. Docetaxel binds to tubulin, inhibiting depolymerization, which ultimately leads to apoptosis via pathways such as the p53 signaling pathway. Prostate tumor cells bearing wild-type p53 are more sensitive to Docetaxel treatment compared to those with mutant or null p53, and down-regulation of p53 significantly inhibits apoptosis induced via Docetaxel treatment.

**Fig 7:**
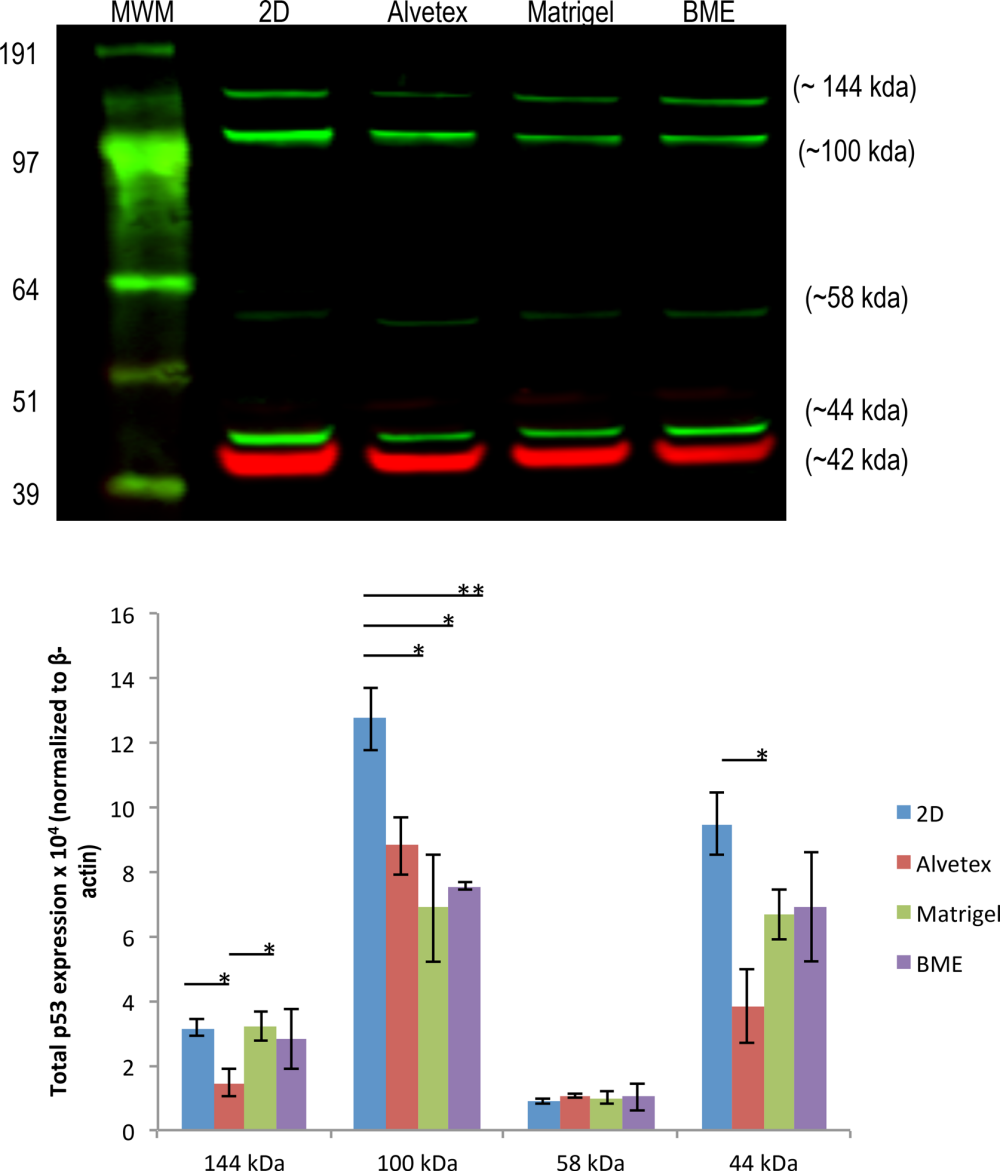
Total EGFR and β-III tubulin protein expression in DU145 cells. A representative image of total p53 protein expression in 2D- and 3D-cultured LNCaP cells. Bars represent the relative protein quantification of p53 isoforms at ~144, ~100, ~58, and ~44 kda on the basis of β-actin, and indicate the mean ± SEM (n = 3 for each group). *p≤0.05 using one-way ANOVA and unpaired t-tests.

Fig 7 shows the expression of p53 in 3D spheroids of LNCaP cells cultured on various matrices, along with that in 2D cultured cells. The western blot showed a band near ~53 kilodaltons, at approximately 58 kDa Most likely, this protein was p53 with post translational modifications [77]. However, this band was not strong and there was no significant difference between the expression of this protein between 3D- or 2D-cultured cells. Unexpectedly, on the blot, there were several other bands detected and were stronger than the band at 58 kDa, possibly because the anti-p53 antibody was able to detect p53 isoforms or p53 proteins in complex with other proteins. As shown in the blot, there are two bands above the p53 band. In *in vitro* studies, it has been shown that p53 monomers (53 kda) first assemble into homodimers, which then go on to form tetramers [78, 79]. p53 dimer was observed at ~100 kDa in some cell lines including non-small cell lung cancer cell line A549 and breast epithelial cell line, MCF10A, when glutaraldehyde was used to induce oligomerization [80, 81]. Based on the protein size, the band at ~100 kDa on the blot was possibly a p53 dimer, although no cross-linking agent was used in our experiment. A further experiment to run the gel with higher concentration of NuPAGE® sample reducing agent did make this band disappear, but still did not enhance the band at 53/58kDa (data not shown). However, it is noteworthy that the expression of this ~100 kDa protein was at a significantly lower level in 3D-cultured LNCaP cells compared to those in 2D culture. Decreased expression of p53 was reported to correlate with an increase in Docetaxel resistance [76]. Therefore, lower expression of the p53 dimer by 3D-cultured LNCaP cells might, in part, account for their increased resistance to Docetaxel treatment compared to cells in 2D culture. In addition, in its active conformation, p53 functions as a tetramer to bind DNA, inducing cell cycle arrest or apoptosis. According to literature, a p53 tetramer is 171–174 kDa [82]. The other band between 97 and 191 kda on the blot was possibly the p53 tetramer; however, this protein seemed to be smaller than the molecular weight of a p53 tetramer. The p53 protein is also known to be able to bind to several proteins [83], therefore, it is possible that this is a p53 oligomer bound to another protein, forming a p53-protein complex[84]. Although the expression of this protein was significantly higher in Matrigel- and BME-cultured cells compared to those in Alvetex, its expression was not clearly correlated to Docetaxel sensitivity of LNCaP cells.

The band below the p53 on the blot at ~44 kDa was likely one of the Δ40p53 isoforms of P53, which lack the transactivation domain of the N-terminus. There are three types of Δ40p53 isoforms: α, β, and γ [85] and all have been shown to influence p53 activity. Δ40p53α has been reported to negatively regulate the transcriptional and growth-suppressive activity of wild-type, full-length p53 [86, 87]; however, β and γ Δ40p53 isoforms have been believed to support the pro-apoptotic function of wild-type p53 by inhibiting MDM2 degradation of wild-type p53 [88, 89]. On the lot, the expression of the Δ40p53 isoforms was lower in 3D-cultured cells in comparison to those in 2D culture. Referring back to the LNCaP Docetaxel response curve, 3D-cultured cells were more resistant to DOC treatment (Fig 4B), suggesting that the expression of β or γ isoforms of Δ40p53 in LNCaP cells is positively associated with LNCaP's sensitivity to Docetaxel.

Nevertheless, in both DU145 and LNCaP cell lines, the influence of matrix on the expression of some of the drug action- or drug sensitivity-related proteins was obvious, and a correlation between the expression levels of these proteins or their isoforms/complex forms and cellular sensitivity to the drugs was observed. Many previous studies reported that protein expression in 3D-cultured cells often differs from their 2D counterparts, which is correlated to the relative sensitivity of the two types of culture to drugs. For example, Kim et al. [90] also showed that glioblastoma cells in 3D matrices displayed increased levels of anti-apoptotic proteins, B-cell lymphoma-2 (Bcl-2) and survivin, which in turn, made them more resistant to apoptosis induced by doxorubicin compared to the 2D monolayer culture. However, very limited studies have focused on the difference among 3D cultures. The results here indicated that protein expression level differed among 3D cell cultured on different matrices and was likely associated with drug sensitivity or resistance, which could be understood similarly to the situation between 3D culture and 2D culture. All together, this highlights the complexity of the current available 3D culture systems and brings up the importance in culture system selection when choosing an *in vitro* model to study cellular behavior and drug screening.

## Conclusions

The findings in this study demonstrated that the matrix used for 3D cell cultures influenced many aspects of prostate cancer cell behavior including morphology, proliferation rate, response to chemotherapeutics, as well as the expression of proteins directly or indirectly associated with drug sensitivity. The extent to which matrix type influenced cellular behavior was dependent upon the cell line, drug's mechanism of action, and protein expression levels in 3D-cultured prostate cancer cells. Cells cultured on the synthetic Alvetex matrix exhibited obvious differences from cells cultured on the two biologically derived matrices (Matrigel and BME) and behaved more closely to cells cultured in 2D monolayer. While 3D spheroids cultured on the two biologically-derived matrices exhibited similar cellular behaviors in general, they still exhibited differences in proliferation rate, varying levels of drug sensitivity, and differential expression of drug sensitivity-associated proteins, which were most likely due to differences in matrix composition such as protein composition and concentrations. Conclusively, our results bring the attention to the influence of matrix in 3D culture systems, and highlights the imperative need to further characterize 3D cell culture models in order to achieve standardization of 3D cell culture technology to be used in high throughput drug screening and cell biology studies.

## Supporting Information

S1 Fig LNCaP proliferation on undiluted and diluted Matrigel and BME. (DOCX)

S2 Fig Proliferation of PC3 cells on Matrigel, BME, and Alvetex, along with in 2D monolayer culture at 72 and 120 h. (DOCX)
