# Supplementary material for: Influence of Matrices on 3D-Cultured Prostate Cancer Cells' Drug Response and Expression of Drug-Action Associated Proteins
Source: PLoS One. 2016 Jun 28;11(6):e0158116. doi: 10.1371/journal.pone.0158116 (PMC4924873; doi:10.1371/journal.pone.0158116)
Supplement: S1 Fig — (DOCX) [file pone.0158116.s001.docx]

**S1 Fig:**  **The Proliferation of LNCaP cells cultured on diluted Matrigel and BME matrices for 120 h.**

Stock Matrigel and BME at protein concentrations of 8.6 mg/mL and 16.03 mg/mL, respectively, were diluted 1:1 and 1:2 with RPMI-1640 medium. LNCaP cells were plated at density of 5,000 cells per well. Cell viability was measured after 72 h using the CellTiter-Glo 3D assay.

For both matrices, increased proliferation of LNCaP cells was observed on the diluted Matrigel and BME compared to undiluted ones. As discussed in the paper, this observation supports that the decreased protein concentrations in the diluted matrix was a possible factor that was related to the increase in cellular proliferation.
