# Supplementary material for: Influence of Matrices on 3D-Cultured Prostate Cancer Cells' Drug Response and Expression of Drug-Action Associated Proteins
Source: PLoS One. 2016 Jun 28;11(6):e0158116. doi: 10.1371/journal.pone.0158116 (PMC4924873; doi:10.1371/journal.pone.0158116)
Supplement: S2 Fig — (DOCX) [file pone.0158116.s002.docx]

**S2 Fig: Proliferation of PC3 cells on Matrigel, BME, and Alvetex, along with in 2D monolayer culture at 72 and 120 h.**

Prostate cancer cell line, PC3 (ATCC® CRL-1435™), an androgen-independent and non-PSA secreting cell line, was purchased from American Tissue Culture Collection (ATCC) (Manassas, VA, USA). PC3 cells were cultured in RPMI-1640 media (Thermo Scientific Hyclone, Logan, UT, USA) supplemented with 10% fetal bovine serum (FBS), 100 units/mL penicillin, and 0.1mg/mL streptomycin (Lonza, Walkersville, MD), in standard cell culture conditions at 37^o^C, 5% CO_2_, and 95% humidity. To culture cells in 3D on Matrigel or BME, the same conditions of culturing DU 145 and LNCaP cells were used for PC3 cells, except an initial density of 5,500 cells/well was used. Cells were left to grow for 72 or 120 h. Cell proliferation was evaluated by luminescent cell viability assay using CellTiter-Glo 3D cell viability assay (Promega Corporation, Madison, WI, USA).

The S2 Fig. shows PC3 cells on Matrgel and BME showed that its growth pattern between 72 h to 120 h was similar to LNCaP cells (PSA secreting), which suggested that the secretion of PSA was not necessarily associated with the increased proliferation of LNCaP 3D spheroids among the biological matrices between 72 and 120 h. Many other factors such as androgen receptor status, expression of matrix metalloproteinases, metastatic characteristics, the growth factors that are able to bind to cell surface receptors to promote cellular growth, may play a role in the proliferation rate of PC cells in various 3D models. Again, the results clearly indicated that the proliferation of prostate cancer cells in 3D cultures was cell-line dependent.
